# Estimating the movements of terrestrial animal populations using broad-scale occurrence data

**DOI:** 10.1186/s40462-021-00294-2

**Published:** 2021-12-11

**Authors:** Sarah R. Supp, Gil Bohrer, John Fieberg, Frank A. La Sorte

**Affiliations:** 1 Data Analytics Program, Denison University, Granville, OH 43023 USA; 2 Department of Civil, Environmental and Geodetic Engineering, The Ohio State University, Columbus, OH 43210 USA; 3 Department of Fisheries, Wildlife, and Conservation Biology, University of Minnesota, Minneapolis, MN 55455 USA; 4 Cornell Lab of Ornithology, Cornell University, Ithaca, NY 14850 USA

**Keywords:** Acoustic monitoring, Camera trap, Crowdsourced data, eBird, Migration, Occurrence data, Population-level movement, Range expansion, Terrestrial animals, Weather surveillance radar

## Abstract

As human and automated sensor networks collect increasingly massive volumes of animal observations, new opportunities have arisen to use these data to infer or track species movements. Sources of broad scale occurrence datasets include crowdsourced databases, such as eBird and iNaturalist, weather surveillance radars, and passive automated sensors including acoustic monitoring units and camera trap networks. Such data resources represent static observations, typically at the species level, at a given location. Nonetheless, by combining multiple observations across many locations and times it is possible to infer spatially continuous population-level movements. Population-level movement characterizes the aggregated movement of individuals comprising a population, such as range contractions, expansions, climate tracking, or migration, that can result from physical, behavioral, or demographic processes. A desire to model population movements from such forms of occurrence data has led to an evolving field that has created new analytical and statistical approaches that can account for spatial and temporal sampling bias in the observations. The insights generated from the growth of population-level movement research can complement the insights from focal tracking studies, and elucidate mechanisms driving changes in population distributions at potentially larger spatial and temporal scales. This review will summarize current broad-scale occurrence datasets, discuss the latest approaches for utilizing them in population-level movement analyses, and highlight studies where such analyses have provided ecological insights. We outline the conceptual approaches and common methodological steps to infer movements from spatially distributed occurrence data that currently exist for terrestrial animals, though similar approaches may be applicable to plants, freshwater, or marine organisms.

## Background

Describing how the locations of individuals or populations change across space and through time is an important part of understanding many different levels of ecological organization. Tracked movements can allow us to understand individual behaviors (e.g., establishing home range, mate seeking, emigration to new territory [1–3]), the consequences of inter- or intra-specific interactions (e.g., competitive, facilitative [4, 5]), how individuals track and acquire resources (e.g., follow resource pulses, seasonality, migration [6–8]), or the effects of natural or anthropogenic perturbations (e.g., relocating from catastrophe or land use change, shifting habitat use in response to climate change or changes in resource availability [9, 10]). Recent technological innovations have expanded our ability to document fine-scale movements, and to track individuals over both short and long distances and time periods. Tracking sensors are becoming smaller, more affordable, and are being applied to an expanding range of taxa (e.g., birds, whales, bats, insects, fish [11, 12]). From such devices, the movements of individuals can be tracked, allowing researchers to connect known locations through time to understand how and where individuals move, or to evaluate the movements of many individuals to understand interactive components of movements among conspecifics or between species, or to summarize how aggregate populations move through time (e.g., [13, 14]).

**Fig. 1:**
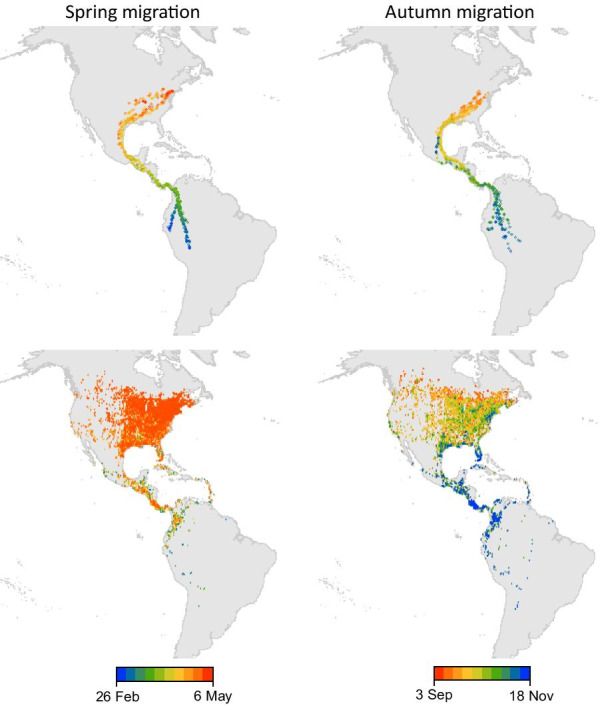
Map of the Western Hemisphere displaying locations of Broad-winged Hawk (*Buteo platypterus*) during spring and autumn migration. The top row shows 7394 locations from 21 GPS-tracked individuals compiled from 2014 to 2020, accessed from Movebank.org (study name “Broad-winged Hawk habitat use, range, and movement ecology, study ID 28691134) on 19 May 2021. The bottom row shows 277, 398 unique Broad-winged Hawk occurrence locations in eBird from 2014 to 2020 [36]

Tracking data, however, are often limited to a small number of individuals (< 30) over short time periods (days to months), restricting the ability of researchers to generate broad-scale inferences [15–17]. In addition, individual-level tracking data are often constrained due to organisms or species having small body size [18, 19], budgetary limitations [20], or high tag loss (anatomical, behavioral, animal safety [21–23]). It can also be challenging to mark and track a sample of individuals that adequately represent broadly distributed species or species with large populations (e.g., [24, 25]). Because of these limitations, it is often more feasible to collect individual locations where identity is not retained over time (occurrence data), especially across large spatial and temporal scales. Even where individual-level movement data exist, complementary approaches that instead use individual occurrence data to study emergent dynamics in population distributions, referred to as population-level movement (previously described in [26–28]), have the potential to address knowledge gaps and advance our understanding of general movement phenomena (Fig. 1), ecological interactions, disease spread [29], invasive species and range-expanding species [30–32], climate response [7, 10, 14, 33, 34], and conservation of mobile populations, such as those that migrate [35].

**Fig. 2:**
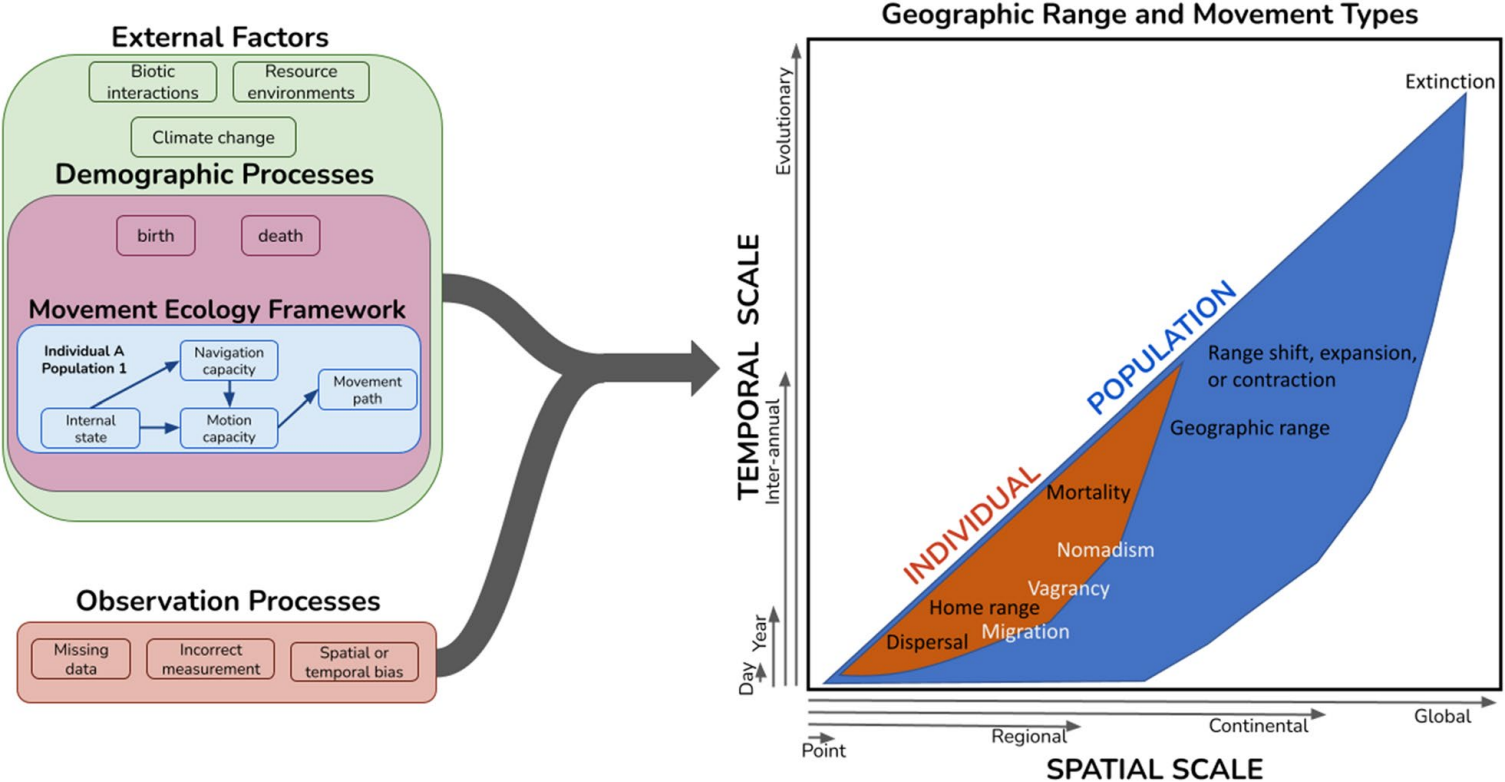
A population-level framework for movement ecology. Measures of population-level geographic distributions and ranges and their quantified movement through time emerge from multiple processes including individual behaviors (sensu the movement ecology framework, [42]) and demographic processes, both of which occur within the context of external factors. Additionally, observation processes may influence observed population-level patterns and must be accounted for to obtain reliable inferences. Population-level movement can be estimated across a broad range of spatial and temporal scales beyond individual-level movement. Population-level and individual-level measures are each capable of capturing movement phenomena with some overlap between approaches such as patterns related to migration, vagrancy, and nomadism

Population-level movement can be summarized by an aggregate metric of the population distribution (e.g., its center or boundary) and quantified by its rate of change (direction, magnitude) across a subset of individuals within a defined population, species, or geographic area. Population-level movement, or redistribution, includes migration, nomadism, and the shifts of previously sedentary ranges or established natal or breeding dispersal areas [26, 37, 38]. Movement at the population-level can result from individual behavior, demographic processes, external factors, or their combined effects (Fig. 2). Despite the strengths of individual-level tracking data, researchers are increasingly able to turn towards occurrence data from human and automated sensor networks to infer macro-scale population-level movements [11, 39, 40]. Recent advancements in data acquisition, processing, and analysis have allowed broad-scale occurrence datasets to be used to infer spatially continuous movement of populations across the landscape over short to long timescales. Adding a macroecological lens to movement ecology provides a novel perspective for connecting individual processes and behaviors to emergent population-level movements across a range of temporal scales (seasonal, interannual, multi-generational, or evolutionary) and the big-picture trends in geographic range movement, expansion, or contraction that are occurring in response to ongoing regional and global changes [41].

Animal occurrence data, often collected as static presence, presence/absence, counts, or density in space and time along with a measure of sampling effort, are increasingly available through widely distributed sensor networks. These sensors can be human, as in the case of crowdsourced initiatives where tasks such as data collection or processing are outsourced to an undefined and large group of volunteers [43]. In other cases, automated sensor networks collect data passively. Examples include weather surveillance radar [39], acoustic monitoring [44], or image monitoring [12, 45]. Some efforts overlap, with automated sensors passively collecting data, through a crowdsourced network of volunteer maintainers (e.g., [46, 47]). In all cases, each occurrence record represents an individual observation, a count, or a density of individuals, but in most cases, records lack individual identifiers that would allow linking the same individual animal to multiple locations at different times, and thus, explicitly record movement. To ascertain movement patterns, general requirements are that the network of sensors be distributed across a broad spatial extent, and that the sensor network collects data at a high enough temporal frequency relative to the movement properties of the species in order to detect shifts in location, and relative to the needs of the research question. For example, to infer population-level movement of a seasonally migrating species, a sensor network would need to include observations from across the summer and winter ranges and within regions of passage, with occurrences documented frequently throughout the species’ annual life cycle (Fig. 1).

Many datasets provide publicly available occurrence location data (Table 1), and the past decade has seen an increase in the number of datasets and new methods developed to infer population movement from occurrence data. Such occurrence datasets are commonly used to estimate species distributions at a single point in time or by pooling data across years (e.g., [48, 49]), which generally represent a static perspective of species location or occupancy across a landscape. Species distribution models are also often used to forecast changes in distribution in response to changes in climate or land use (e.g., [50, 51]). While species distribution models do not directly estimate movement, they can be used to measure different aspects of a population’s location in time and space, and by linking changes in those measures across time, one can infer patterns in population-level movement that include migration, nomadism, or range shifts. To date, little synthesis has been done on the data, methods, and types of models used to infer population-level movement from occurrence data. The purpose of this review is to (1) to lay out a theoretical framework to understand and guide population-level movement research, (2) summarize existing broad-scale species occurrence datasets, (3) discuss the latest statistical and modeling approaches for inferring population-level movement, and (4) highlight where such studies have provided ecological insights. Through the review, we hope to provide guidance to researchers conducting their own movement analysis using occurrence data, and to identify areas ripe for future research or development. We restrict our discussion to population-level movements in terrestrial animals, mostly at regional and larger spatial scales, and seasonal or longer temporal scales, though similar insights may be applicable to marine or aquatic animal studies, or at finer spatial or temporal resolutions (Box 1).

**Box 1:** Key terminology needed to use this review as a guide

Glossary	
*Tracking data* Locations of uniquely identified individuals that are linked through time *Occurrence data* Locations that cannot be identified to specific individuals but can be labeled as belonging to a particular population, species, or taxonomic group. Occurrence records at a location may be measured as presence, count, or density values *Population* The group of individual observations to which one wants to make inference. For example, a subset of individuals within the same or multiple species, a subpopulation within a defined geographic area, a meta-population considered as a whole across a region, or even a whole species spread across a region or continent. This definition differs somewhat from a biological population, and is more similar to a statistical population, which is defined by the set of observations of interest for a specific question *Individual-level movement* A movement path generated by linking locations of the same individual through time *Population-level movement* Population redistribution over time, which can be summarized by an aggregate metric such as center or boundary, and quantified by its rate of change in direction or magnitude. Movement at the population level can result from individual behavior, demographic processes, external factors, or their combined effects *Crowdsourced data* Data collected with or without strict protocols by volunteers distributed across many locations, and placed into a repository for review and inclusion in an overall database. Advanced internet technologies are often used to harness these efforts. May also be referred to as citizen science, civic science, community science, or public monitoring data *Structured to unstructured data continuum* Structured data are typically stored in tabular or relational database formats, machine readable and could be readily used in an analysis. In contrast, unstructured data are typically found in audio, image, video, or unstructured text formats, are not readily machine readable and require further specialized processing to be ready for analysis. For example, conversion and translation are needed to interpret the raw data (i.e., a target that is visible in an image or audible in a recorded sound) to an identification of the presence of an individual of a particular species. Semi-structured data fall somewhere in between, for example in xml formats where user-defined tags may be used [52] *Structured to unstructured project continuum* Structured project or network designs collect data with rigorously prescribed protocols and tightly controlled measurement error, ideally with randomization to ensure representation of the overall population, and are implemented for a specific purpose or planned data analysis with clear objectives. In contrast, unstructured projects or network designs collect data by open recruitment, with few rigorous protocols, and with typically large variation in data quality and quantity within the network. Semi-structured projects fall somewhere in between, for example, by collecting information on potential covariates or biases that can be accounted for in later analysis (sensu [53, 54]) *Movement Ecology Paradigm* Nathan et al. [42] proposed this paradigm to organize individual movement research, based on four mechanistic components of organismal movement: (1) internal state, (2) motion, or (3) navigation capacities of an individual, and (4) external factors affecting movement	

## A theoretical framework for population-level movement

Recent statistical and modeling advances have allowed researchers to use occurrence data to infer spatially continuous population-level movement across the landscape. The types of population-level movement questions that can be answered with occurrence data differ from traditional individual-level movement questions using tracking data. Occurrence data are well suited for documenting movements between regions that occur in a relatively short time frame, such as days, weeks or months (migration, nomadism, or dispersal; sensu [17, 37, 38]) or for documenting movements and geographic range shifts that occur across a relatively long time frame, such as multiple years, generations, or evolutionary time [55, 56]. In contrast, occurrence data are poorly suited for determining the movements within resident populations (e.g., encamped movements related to behavioral activities like foraging). Beyond describing a movement path, the movement ecology paradigm identified three basic components focused on individual movement—internal state, navigation capacity, motion capacity—and their correlation with external factors [42]. Importantly, since occurrence data frequently represent locations where individual identity is not retained over time, movement paths cannot be described for specific individuals, and variation in movement cannot be attributed to individual characteristics such as age, genetics, phenotype, behavior, or interactions [57]. Instead, occurrence data can be used to identify population-level movement patterns that emerge from demographic processes and the movement of individuals. Population distributions can be compared within or across years or between different groups (i.e., species or regions), and changes or differences in distribution may be used to discern external abiotic or biotic correlates of movement.

We outline a population-level movement framework that builds upon the individual-level movement ecology paradigm [42], recognizing that population-level distributions emerge from individual movement mechanisms [26] and movement types [38] along with demographic processes (Fig. 2). This framework recognizes the disparate spatial and temporal scales at which the individual- and population-level processes often play out and organizes different movement types within these scales. Emergent changes in population distribution (i.e., population-level movement) may be influenced by scale-dependent external factors, including biotic interactions, resource environments, climate change, and anthropogenic disturbance [26, 38]. In some cases, such as migration and nomadism, the different types of population-level movement roughly overlap with types that can be observed in individuals [37], whereas other types of population-level movement, such as range shifts, emerge from aggregate, long-term changes in individual behavior and demographic processes.

## Research themes

We propose grouping population-level movement research into four thematic research areas: (1) Quantify population-level patterns of movement, (2) assess how species traits influence population-level movement (i.e., internal factors), (3) study how population-level movements correlate with external factors, and (4) connect movement patterns with conservation or management schema (i.e., implications and applications) (Box 2).

**Box 2:** Thematic research areas and specific research questions that are important to the emerging field of population-level movement ecology

Example research categories and question types	
*Research Theme 1: Quantify population-level patterns of movement*	
1. How does the geographic center of a population change seasonally and through time? What is the distance covered, rate of temporal change or speed, directionality, and intra- and inter-annual variation? What is the timing of migration and how does the distribution of a population change during migration? 2. How does the location of range boundaries or population clusters within a species’ range, change seasonally and through time? 3. How does the population’s movement compare to other populations or species?	
*Research Theme 2: Assess how species traits influence population-level movement*	
4. How is population movement constrained or facilitated by average behavioral, physiological, or morphological traits of the species? 5. For migratory species, how do migration strategies (e.g., partial, full, differential, irruptive), migration distance, morphology (e.g., body mass), or behavior (e.g., diet) impact movements? 6. To what extent are observed differences among species explained by their traits?	
*Research Theme 3: Study how population-level movements correlate with external factors*	
7. Which external factors (ecological, environmental, geographic, or anthropogenic) correlate with population-level movement? How and where do populations move in relation to these external factors? 8. What are the most relevant spatial and temporal scales for biotic or abiotic interactions to impact movement? 9. Can we develop empirical mechanistic models of population-level movement based on the observed occurrencees and external factors?	
*Research Theme 4: Connect movement patterns with conservation or management schema*	
10. How does the population’s movement or the movement of it’s range center or edges contribute to or change biodiversity patterns? 11. What environmental or landscape factors are needed to maintain or improve population movement efficiency or to reduce risk during movement? How are the consequences of global change (climate change, land-use change, and environmental pollution) affecting, or forecasted to affect, population-level movements? 12. Do movement trends and associations with environmental drivers suggest changes to location or range that could help guide priority concern or management strategies? Are there natural or anthropogenic barriers to movement that might be important when considering conservation under changing climate, where species may seek to move to colder areas at higher latitudes or elevations?	

These categories originate from the new population-level movement framework proposed here, and the constraints that limit certain types of analyses when individuals identities cannot be retained

## Occurrence data

Most sensor-network occurrence data fall somewhere along the continuum of structured to unstructured *data* [52], and of structured to unstructured *projects* (Box 1, [53, 54, 58]). From a big data perspective, occurrence *data* generated by human or passive automated sensors may be structured as tabular or database formats that are easily machine readable (analysis ready), unstructured as audio, image, video or text files that are not easily machine readable, or fall somewhere in between as semi-structured data [52]. From a study design perspective, *projects* that generate occurrence data range from structured to unstructured sensor networks. Structured projects use rigorously prescribed protocols, implement systematic or random sampling to ensure locations are representative of a larger population of interest, and are implemented for a specific purpose or planned data analysis with clear objectives [53, 54], for example, long-term standardized projects like the UK Butterfly Monitoring Scheme [59] or national weather surveillance radar systems [60]. In contrast, unstructured projects are collected by open recruitment, with few rigorous protocols, and typically exhibit large variation in data quality and quantity (e.g., iNaturalist, [61]). Semi-structured projects fall in between [53, 54, 58]. Data resulting from semi- and unstructured projects are not typically designed to answer specific research questions [53], or to adhere to particular statistical or study design principles but are often collected with the goal of sampling a large portion of the total population within a defined area [40]. Unstructured and semi-structured project examples often include human sensor networks such as eBird [36] and eButterfly [62], or other crowdsourced data platforms relying on professional or volunteer observers [43, 63] that may or may not collect effort covariates designed to account for potential sources of bias in the data [58, 64]. Structured project examples often include passive automated sensor networks that are designed by a central institution (e.g., weather surveillance radar as part of a national weather monitoring system). Structured projects may collect structured, semi-structured, or unstructured data, and the same is true for unstructured projects. The proliferation of occurrence datasets and projects that vary from structured to unstructured provides new challenges and opportunities for observing and estimating population-level movements across broad spatial scales.

Crowdsourced data can leverage the interests or expertise of many individuals to collect relatively high-density occurrence data across broad geographic areas and at high frequency through time. Many such efforts have gained traction with general community members and provide rich, and often publicly available, data sources [65]. The applications or websites on which volunteers enter occurrence information can provide some structure to the data, while allowing for flexibility in observer expertise and motivation (e.g., a casual observer who largely ignores protocols to an observer that is dedicated to following strict protocols). Many platforms for crowdsourced data provide a method for “vetting” or filtering the data to address obvious data quality issues such as misidentifications. eBird (www.ebird.org) has been a leading example, capitalizing on already dedicated birding groups and hobbyists, and developing a platform that mimics the checklist format already popular among birdwatchers [36]. New analytical methods and procedures have been developed to leverage the information provided by eBird to generate reliable estimates of species occurrence [66]. Other datasets focus on different taxonomic groups and geographic regions, but are increasingly providing the quality, density, and frequency of human-observed data necessary to assess population-level movements (Table 1). Appropriate use of crowdsourced data requires careful consideration of imperfect and variable detections as well as spatiotemporal variation in sampling intensity, with observations often biased towards easily accessible locations containing unique or abundant species [64, 65, 67–69].

**Table 1:** Examples of occurrence datasets that are publicly available or can be accessed through a registered user account

Example occurrence dataset	Sensor type	Taxa	Spatial extent	Temporal extent
iNaturalist; https://www.inaturalist.org [61]	Crowdsourced human observers	Any	Global	2008–present
Global Biodiversity Information Facility (GBIF); https://www.gbif.org/ [70]	Professional, governmental, and crowdsourced human observers	Any	Global	2001–present
eBird; https://ebird.org/ [36]	CROWDSOURCED human observers	Birds	Global	1800–present
Herpmapper https://www.herpmapper.org/ [71]	Crowdsourced human observers	Herptiles	Global	2013–present
eButterfly; https://www.e-butterfly.org/ [62]	Crowdsourced human observers	Lepidoptera	North America	2011–present
UK Butterfly Monitoring Scheme; https://ukbms.org [59, 72, 73]	Volunteer, professional, and governmental human observers	Lepidoptera	United Kingdom	1976–present
United States weather surveillance radar [74, 75]	Weather surveillance radar	Aerofauna	North America	1991–present
European weather surveillance radar; OPERA [60, 76]	Weather surveillance radar	Aerofauna	Europe	2012–present
North American Bat Monitoring Program; [77] https://www.nabatmonitoring.org/	Professional and governmental acoustic surveys	Bats	North America	2009–present
Snapshot USA (eMammal); https://emammal.si.edu/snapshot-usa [55]	Crowdsourced camera traps	Terrestrial mammals	United States	2019–present
FrogID; https://www.frogid.net.au/ [56, 78]	Crowdsourced human observers via acoustic app	Frogs	Australia	2017–present

The temporal extent is noted for each dataset, though it is important to recognize that most of these efforts have a significant “ramp up” period, and the frequency and quality of data from the earliest years may not be high enough to support broad-scale analyses. This list is not exhaustive and is meant to illustrate different taxonomic examples across the globe that could be used to infer population-level movement

In addition to the crowdsourced data provided by direct observations, new technologies are expanding opportunities for automated observation networks, which can be used to document occurrences and infer population-level movements. Examples of automated networks include weather surveillance radar (WSR), acoustic monitoring, and camera traps. WSR stations were developed for the purpose of monitoring precipitation, but such data streams provide new opportunities to monitor biological populations (e.g., [79, 80]). These data may be used to monitor specific taxonomic groups (e.g., birds, bats, or insects) but the data typically cannot be parsed into individual species [81–83]. Exceptions include species that occupy large roosting sites during the night, whose dawn departures can be detected by WSR [84, 85]. Significant technical knowledge is often required to screen and process WSR data [86]. After processing, WSR data may be capable of providing relative densities of biological targets as altitudinal profiles of density, speed and direction [87, 88]. Like crowdsourced occurrence data, WSR data cannot track specific individuals, but it can provide a cost-effective density-based estimate of the distribution and movement of aerofauna across space and through time. There are several national and international WSR networks that provide data openly, or in agreement with specific research groups. WSR data is freely available in the United States (i.e., [74]) and in Europe through a multinational data exchange program (OPERA, [60, 76]). Although some data and methods for parsing biological entities have also been published (e.g., MistNet, [89, 90]), acquiring and analyzing WSR data across other geographic and political regions may be more challenging due to WSR coverage gaps, limited data accessibility, and interoperability of data streams across stations [80, 91, 92].

Automated sensor networks may also include audio or visual technologies, such as acoustic monitoring and camera traps, and are sometimes deployed or maintained using crowdsourced volunteers (e.g., [46, 77, 93–95]). Camera trap and acoustic sensors may vary in their ability to isolate likely image or audio targets, may be patchily distributed across the landscape, and users may need to rely on automated software tools that are not 100% accurate or require time-consuming manual vetting processes [96]. Thus, one challenge with acoustic and camera trap data is the combination of technical software and skill needed to identify and isolate the correct sounds or images for analysis [97], but also the human time that is often needed to manually validate portions of the data for accuracy [96, 98, 99]. Sparse arrays of acoustic or camera monitors may be useful for confirming a species’ occupancy, or for estimating animal activity patterns, abundance, or species diversity in an area, especially when robust methods for confirming species presence have been developed [100, 101], but much larger and denser arrays would be needed to infer population movement through or within an area [46]. Differences in camera trap survey designs, including baited versus unbaited stations, have been found to have significant consequences for occurrence frequency and detection rates [102, 103]. However, there are several examples of such arrays that have been used to infer population movement—acoustic recordings for bat occupancy trends across space and through time [104], camera traps for raptor prevalence and migration [46] and migration timing and speed of caribou and ptarmigan [105]. Although acoustic monitoring is most frequently used to observe species within a local area or during non-movement periods, it has also been used to detect bird populations during migration [106] or to “catch” the short flight calls that birds emit during migration [107]. Decreasing costs of Autonomous Recording Units (ARUs) and camera units may increase the feasibility of deploying these sensor technologies to detect and infer population movement in future studies [100, 108].

Due to the partially unstructured nature of data, common for all of these networks, these datasets require a significant time investment to process and validate data, and careful consideration of possible sampling biases and variability in an observer’s skill before they can be used for analysis. A benefit for population-level movement research is that much of these data are publicly available (Table 1) or are available upon request, in contrast with individually tracked data, which may be more likely to be protected and only accessible within specific labs or institutions (but see Movebank’s data repository for a library of openly published individual track data; https://www.datarepository.movebank.org/). Data from crowdsourced human and sensor networks are most readily available at appropriate densities from the northwestern hemisphere, specifically from North America and Europe, and have a strong bias towards aerofauna, including birds, flying insects, and bats.

## Analytical approaches to estimate population-level movement

### Data processing

**Fig. 3:**
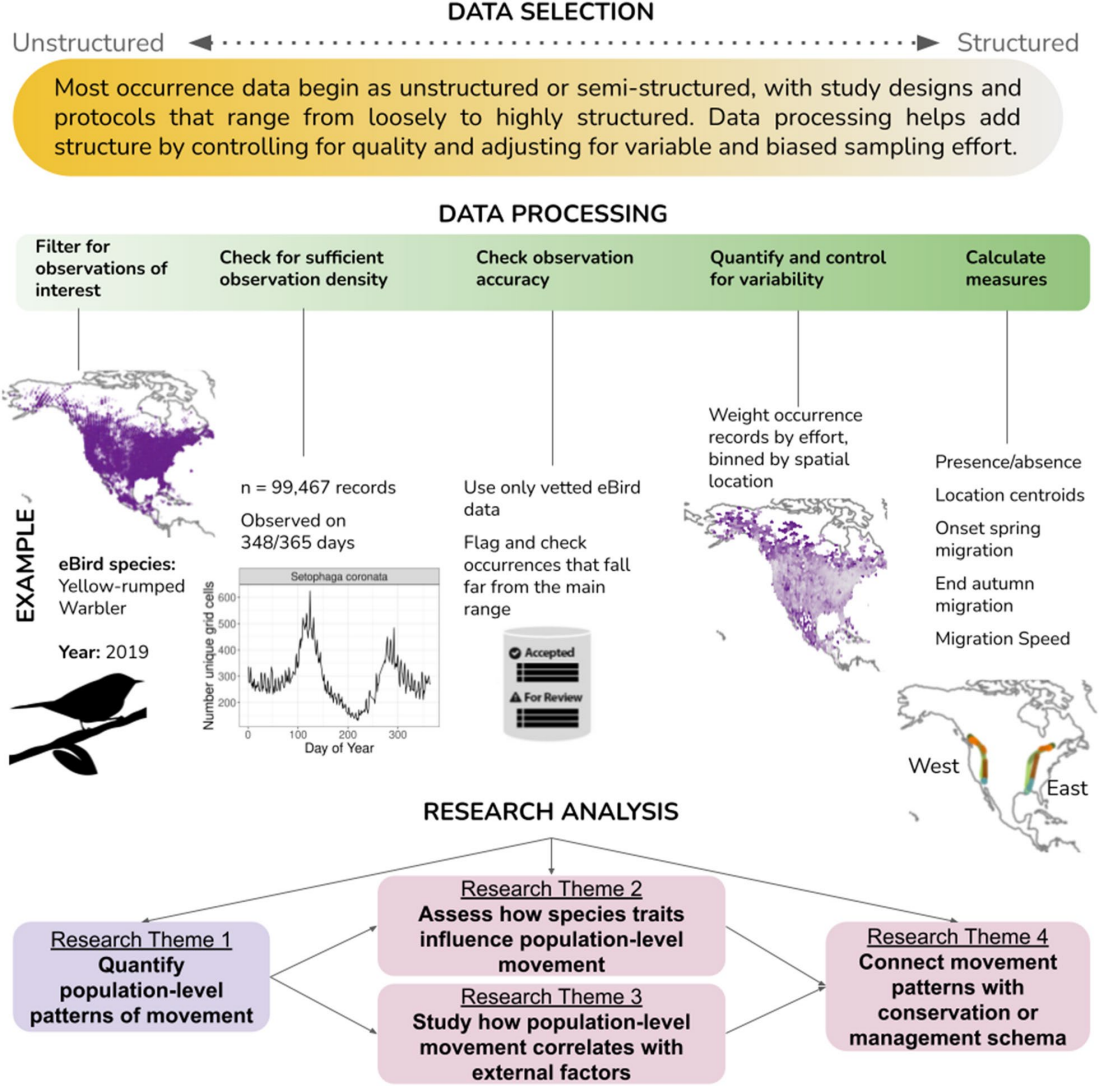
Schematic of the steps from data selection to data processing and analysis that could be used to evaluate population-level movement from occurrence data. An example is shown using eBird occurrence data from the western and eastern flyways of the Yellow-rumped Warbler (*Setophaga coronata*) in 2019 [36], but the same general workflow could be applied to other occurrence datasets. Yellow-rumped Warbler silhouette was created by Cornell Lab of Ornithology and is used with permission

Prior to statistical analysis, occurrence data frequently require cleaning and processing. Data processing methods may differ among datasets and for distinct research questions, but general challenges include estimating occupancy from presence-only data [109], standardizing sampling intensity by subsampling observations (e.g., [67, 110]), accounting for low detection probability of certain species or in certain time periods or habitats [102], the potential for false positive occurrences [96], and accounting for detection or sampling biases related to human behavior [66]. Clear guidelines or code to aid in processing the data may be available for some datasets, or may require more technical knowledge to navigate, especially for WSR, acoustic, or image data [89]. In other cases, common clear methods may not exist, and a researcher may need to develop their own data processing workflow using appropriate analysis techniques that account for imperfect detection or variable sampling effort (Fig. 3).

### Data vetting and cleaning

Technical and logistical challenges exist for researchers using semi-structured or unstructured data, as data are often not ready “out of the box” for analysis, and because different data and project structures require unique statistical approaches to minimize bias [53, 111, 112]. Each occurrence dataset will contain different fields, constraints on data collection, variability among sensors or observers, and the oversight or protocols behind a given project. These factors need to be accounted for to reduce bias and avoid flawed conclusions (Fig. 3) [58, 65]. Variability in data collection and data quality can often be dealt with in the data processing stages but may also need to occur within the data analysis stage. When preparing occurrence datasets for movement analysis it should be useful to follow some or all of the checks presented below for filtering data, checking for coverage, accuracy, and accounting for variability.

### Filter for observations of interest

For any occurrence dataset, an initial first step is often to parse out observations that represent biological entities, the target specie(s) and/or locations of interest, and observations that have a high likelihood of being accurate (e.g., not a misidentification). For WSR and other automated sensor network data such as acoustic or camera arrays, simply identifying records that represent biological entities, and then filtering to the target species or taxa is itself a non-trivial process (e.g., [113, 114]). Many tools have been developed to screen and process WSR data for biological information including the Bias Improvement of Radar Data System (BIRDS; methods described in [115]), and computational packages such as w2birddensity (part of WDSS-II [116]), vol2bird [87], R package bioRad, [117], and MISTNET [89]. Camera trap and acoustic data research also increasingly rely upon artificial intelligence systems to automate and speed the process of filtering for target biological entities [97, 118–122], possibly using image or acoustic libraries (e.g., [123]). But such data still often require a significant investment in manual human-verification (e.g., [98]) before the data can be used for analysis.

### Check for sufficient observation density and trends over time

**Fig. 4:**
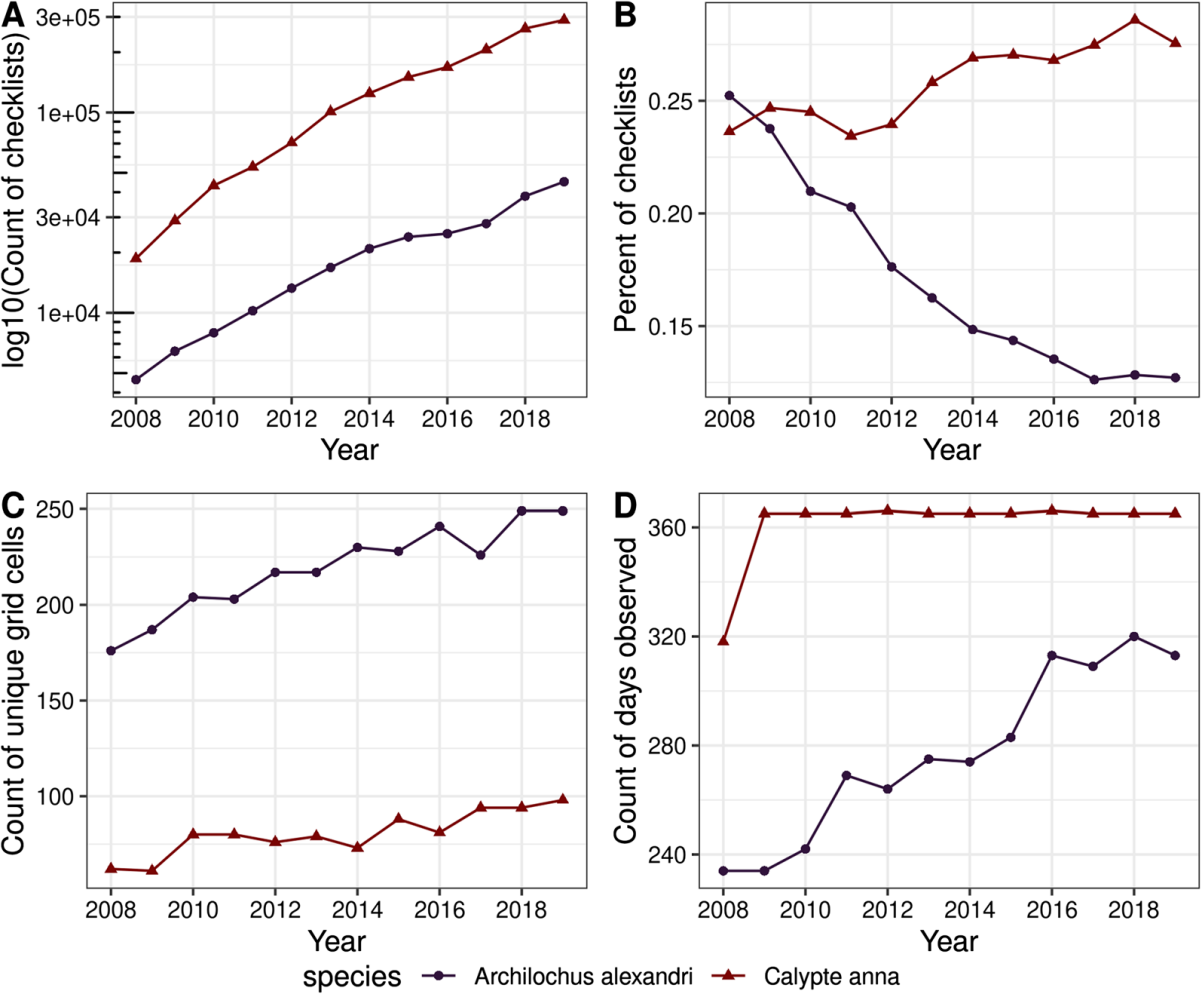
A worked example exploring observation trends in eBird occurrence data from 2008 to 2019 for two closely related species: migratory Black-chinned Hummingbird (*Archilochus alexandri*) and range-expanding Anna’s Hummingbird (*Calypte anna*) [125] from the western flyway of North America [36]. Even closely related species can display different dynamics, which can dramatically affect how the data is structured across space and time. (**A**) The total number of checklists (log_10_ transformed) containing each species increases through time, which is expected as the crowdsourced platform gains new observers. It does not represent an increase in the total number of hummingbirds. (**B**) In contrast, the percent of all checklists containing each species within regions where each occurs is declining for Black-chinned Hummingbirds, and increasing for Anna’s Hummingbirds, which may reflect changes in observer behavior, expertise, or geographic coverage through time. (**C**) After spatially binning the data, the number of unique grid cells in which each species is observed increases slightly through time, but is relatively flat in recent years, giving some confidence that the species’ locations have been adequately covered through the time frame and within the spatial area. (**D**) The number of days that the species was observed in each year is flat for Anna’s Hummingbird after 2008, indicating that they were observed every day in each subsequent year. In contrast, Black-chinned Hummingbirds show a strong increasing trend, which suggests a need to further explore the data to see whether it indicates increased observer effort in general, or at particular locations or times of the year, or if it represents a meaningful ecological trend in the occurrence phenology of the species

To infer movement, the data need to have adequate coverage across space and time. Determining if there is sufficient data can be done in multiple ways, but often requires simply exploring patterns or structure in the data across time and space (e.g., using binned data). Simple visualizations or descriptive summary statistics may help the researcher determine if patterns occur due to imbalances in sampling, survey effort, or data collection methods, or represent valid ecological patterns. Strong increasing trends in total occurrences, the number of spatial locations observed, or the number of time frames observed may indicate that a data set is undergoing strong growth and should be used to infer movement with caution, or that it requires the application of a subsampling or weighting method as a first data processing step (e.g., [124]) (Fig. 4). Less pronounced increases or plateaus in the occurrence trends may indicate more stable data collection across space and time (Fig. 4). Controlling for overall sampling effort, spatially or temporally binning occurrences, and resampling or subsampling methods may all be useful ways to control for variability in sampling effort when analyzing occurrence data. In some cases, the researcher may decide to drop certain time frames or spatial areas from analysis if they do not meet a minimum threshold of occurrences for analysis (inclusion criteria; e.g. see methods from [124]).

### Check observation accuracy

Data veracity is a challenge for many occurrence datasets, and substantial variability in data quality may exist, including the existence of false positive or false negative records. Such inaccuracies, misidentifications, or imperfect detections are mainly a problem if they change significantly over space or through time. Fortunately, many occurrence datasets have protocols in place for human experts to validate questionable observations. For example, programs like eBird, Project FeederWatch, and HerpMapper use automated processes [126] and expert review [36, 71] to validate the data. Expert review may also be used to validate signals resulting from automated classification processing of acoustic or camera sensors [98, 99]. When using occurrence data, a researcher should first review the guidelines for individual datasets (e.g., [66, 127]) or view protocols or methods from recent publications that address potential sources of inaccuracy in the data (e.g., false-positive occupancy models [96]). When guidelines do not yet exist, researchers may need to rely on expert knowledge to look for and filter outliers or suspect records on their own. A researcher may want to consider the species- or habitat-specific context for their research subject and use their own protocols to filter occurrence records outside the known species range or cases where the species is known to have low detection probability or high misidentification rates (e.g., in the case where two similar species co-occur). Often, occurrence data are used to quantify occupancy (presence within a spatial area or grid cell) or relative abundance. Although abundance estimators are sometimes used with occurrence data, these methods often require strong assumptions (e.g. population closure, no double counting, and constant detection probability) that are difficult to verify and can significantly impact estimator accuracy [128–130].

### Quantify and control for variability

Perhaps the largest challenge is to acknowledge and account for sources of variability that originate from the observation process (e.g., variability in sampling effort or observer skill level) within occurrence datasets and control for it where possible. Substantial variability in data quality, data volume, and overall sampling effort across time and space are characteristics of “big data” [52, 65] that are also common to occurrence data. Data fields that record sampling effort, sensor placement, factors related to detection probability, and spatial and temporal resolution of the data, should be collected and included in models to help control for variation when possible. Other effort-based measures may be used, such as time spent searching, distance traveled while searching, number of observers in the search party, or time of day (e.g., eBird [36]). For camera traps or acoustic sensors, effort-based measures could include fields for the number of days a camera/acoustic sensor was active, whether it was baited or not, or if sensors were placed randomly or chosen opportunistically to increase detection, for example, by focusing on known travel routes or previous occurrence locations [103]. Such measures can help to control for crowdsourced effort differences across time and space, rather than using the raw occurrence data.

Recent work has focused on developing methods and approaches that account for variable and spatially biased sampling effort, sometimes by integrating data from multiple structured and/or unstructured projects [131–133], binning the data spatially and/or temporally ([28], e.g., [134]), or weighting records [135]. For example, data can be standardized within a spatial bin by accounting for overall sampling effort (e.g. the total number of observers). Effort measures can be used to weight records, as predictors in an analysis, or in some cases, modeled using an offset (a predictor with a regression coefficient = 1, so that a count-based model effectively models density or encounter rate, [136]). In some cases, rarefaction or resampling methods (i.e., oversampling or undersampling to a median value [124]) may be used to represent a similar sampling effort across space or through time. Variable sampling effort over space is often accounted for using covariates that are suspected to correlate with sampling effort, such as distance to the nearest urban center (e.g., [137]) or distance to road [68]. In some cases effort covariates do not exist (e.g., iNaturalist [101]) and researchers must instead control for variable sampling effort with other methods, such as using the number of non-target species detections as a way to estimate change in effort across space and time [138]. Another common approach used when fitting species distribution models is to sample background locations in a way that attempts to mimic sampling biases in the occurrence data [139, 140]. Alternatively, setting strict inclusion criteria may help to deal with variability by omitting extreme or outlier observations or spatial or temporal bins that do not meet a predetermined threshold for total number of occurrences.

Ultimately, variation in data and project structures makes interoperability across datasets challenging. A major goal for researchers should be to identify better ways to coordinate data collection efforts, to link data observations across different collection sites and platforms, and to develop automated tools to make this process accessible to researchers at different computational skill levels and from different subfields. Species distribution modeling represents one area of research that has developed methods to integrate different types of occurrence data (e.g. presence-only, presence/absence, and count), that might prove useful for aggregating occurrence data to infer population-level movements or the causes of those observed movements [131–133, 141, 142]. Improved standards for data collection and project structure, and adherence to data sharing policies across institutional, national, and international boundaries would benefit the utility of occurrence data for movement ecology [80], considering that animal movement research often requires monitoring at regional, continental, or global spatial scales.

## Methods and models

### Statistical methods

Population-level movement can be inferred from occurrence data in multiple ways: (1) A summary of predicted occurrence distributions at multiple points in time can be modeled to evaluate change (e.g., in the center or boundaries) over time, and (2) changes in the occurrence distribution can be directly modeled as a function of temporally changing explanatory variables. Statistical processes for inferring population-level movement from semi- and unstructured occurrence datasets and projects must be able to account for a high volume of data that contain variable quality, noise, recording anomalies, spurious correlations and incidental endogeneity throughout [52, 143]. Incidental endogeneity is a genuine relationship between predictor variables and the error term in a regression analysis (i.e., residual term is dependent on some of the predictors), and is common in observational data and in highly dimensional data that comes from multiple different sources, such as crowdsourced data [52, 112, 143]. Occurrence data also frequently violate common statistical assumptions of independence, stationarity, and Normality [40]. Spatial [144–146] and temporal [147, 148] autocorrelation are common concerns. Additional challenges include finding ways to deal with multicollinearity (i.e., when an explanatory variable can be predicted by linear combinations of other explanatory variables [149, 150]) or overfitting due to excessive model flexibility (e.g., too many predictors or too flexible a relationship between predictor and response varaible) and accidentally masking the true effects. Generalized additive models (GAMs), and tree-based and other machine-learning methods use cross-validation or penalization in an attempt to avoid overfitting. Even in the absence of multicollinearity and overfitting, models, including advanced tree-based or machine-learning methods, may still have low predictive ability when transferred to novel locations or scenarios [151]. Spurious correlations can occur, particularly when attempting to identify important species traits and environmental drivers of population-level movements with large datasets [143]. Measurement errors can compound, particularly when predictor variables are remotely sensed and are thus only available on coarse grids or where they are spatially or temporally mis-aligned with occurrence records. Based on the above challenges and assumptions, there is a strong need to present levels of uncertainty associated with movement models based on occurrence data.

Modeling approaches should be tailored to the data and the specific research theme and question of a given project. Some modeling approaches use only occurrence records to describe movement paths or trajectories after vetting and controlling for bias in the occurrence data itself (*Research Theme 1*). Others rely on additional information such as species traits, behavioral models, environmental covariates, or anthropogenic factors (*Research Themes 2–4*). Below, we summarize recent analytical methods and approaches for describing or quantifying population-level movement across the four research themes, with a focus on methods for *Research Theme 1*, describing population-level movement.

#### Research theme 1: quantify population-level patterns of movement

Quantifying population-level movement is an important step to integrating population-level and landscape-scale perspectives into movement ecology, which to date has largely focused on the movements of individuals. Crossing or combining organizational scales to understand the changes to population-level measures of distribution that emerge from individual processes is a growing area of study that can provide new insights. Due to the nature of occurrence data, population-level movement is usually characterized using quantitative measures of the population’s range area, range edges, or range center, and changes in these characteristics through time. For populations undertaking directional movement through migration, or undergoing range expansion or range shifts, measures may describe location, direction, density of moving individuals (migration traffic rate, [152]), or speed (e.g., [28, 105]) over a time period, or compare differences in location, density, or velocity across multiple time periods and across space (e.g., [153, 154]). Estimates of the timing of events, such as date of first arrival, crossing a latitudinal or longitudinal demarcation, or reaching a predetermined number or density of observations (e.g., date when half the population has passed a sample point [105, 155]), may also be used to infer or evaluate population-level movement.

One may characterize the central location and movement of a population using coarse summaries of the occurrence data, such as the population’s centroid in latitude and longitude for a given time period (e.g. daily or weekly). The population centroid can then be compared across time or thresholds that indicate movement [28, 105] or arrival at a predetermined area (e.g., [155]). Changes in centroids can be modeled using generalized additive mixed models (GAMM, [156]) or any regression-based approach, allowing users to estimate when migratory species begin or end directional movement (e.g., the onset of spring migration or the end of autumn migration). Further, measures of distance, speed, and direction of movement across seasons or years can be calculated from measures of population centrality. For example, population migration speed may be calculated by measuring the distance between daily centroids as km/day (birds 28) or km/hr (caribou 99) traveled. For longer-term occurrence datasets, these approaches are able to provide a description of population movement through annual life cycles, while accounting for variability among years using random effects (e.g., [134]).

Robust adaptive spatio-temporal models (AdaSTEM), a form of ensemble species distribution models, have been developed for use with eBird data. These models automatically select the appropriate sized stixel (spatio-temporal block of data) for inferring occurrence and abundance across a region based on the quantity of observations [124, 157]. These models do not measure movement, per se, but their estimates of occurrence, abundance, and range can be compared to evaluate how populations move based on shifting species distribution ranges and centers over the target time period. AdaSTEM models are useful because they are semi-parametric and can be used to generate hypotheses for migration dynamics and range expansion, or dispersal across dynamic species ranges, as is necessary especially for migratory species [67]. Estimates of ranges can be compared across different times of the year, or across years, to infer population movement [124]. Scientists working with eBird data have led the development of many new statistical methods and tools for conducting movement research using occurrence data. These new approaches have been successfully applied to eBird data, which contain a large amount of high quality, vetted occurrence records for birds, across broad geographic areas for more than 10 years [36, 66]. Nonetheless, some aspects of these eBird-inspired workflows and statistical methods could be reasonably adapted to other crowdsourced or occurrence datasets, different taxonomic groups, and other types of population movement research questions.

Rather than use a two-step approach (estimating species distributions at different time points and then modeling summary measures of these distributions, e.g. their centrality), it is appealing to consider more mechanistic approaches that model movement in terms of an advection–diffusion process [158]. This approach has been successfully applied to model the spread of Eurasian Collared-Dove (*Streptopelia decaocto*) using structured breeding bird survey data [55], but we are unaware of any successful applications of diffusion models to unstructured project survey data. The modeling approach of Wikle [158] is an example of a state-space model, with separate models for the underlying biological movement and observation processes. Alternative formulations of state-space models could be considered for quantifying population-level movements, but these methods require a significant understanding of advanced mathematics and access to high-level computing tools or high-performance computing systems to fit these models to large datasets [159].

#### Research themes 2 and 3: evaluating the effects of species traits or external factors on movement

Once population movement has been characterized, a logical next step is to assess possible traits or external factors driving movement Outputs from the previous step that describe population-level movement can be used to examine predictors or correlates of movement and to test relevant questions or hypotheses. Since population-level movements are often summarized using an aggregate measure of the population (e.g., its center or range boundary), predictors should be expected to influence a significant number of individuals across a large spatial or temporal scale. Predictor variables frequently come from other datasets, for example, remote sensing products (e.g., NASA MODIS, LANDSAT, landcover or anthropogenic databases, other species occurrence records) or climate reanalyses (e.g., Daymet, NCEP, ECMWF, ERA5), or trait databases [160–162]. These predictors can be analyzed with population-level movement metrics using generalized linear mixed models that account for different sources of variation in the data using combinations of random and fixed effects [163, 164]. In some cases, machine learning based models (i.e., random forests) may be able to identify the relative importance of different predictors for movement and/or changes in occurrence (e.g., [154]).

#### Research theme 4: connect movement patterns with conservation or management schema

Most conservation applications will apply an understanding of how distributions and movement are influenced by species-level or environmental covariates to a particular location or set of locations. In other words, *Research Theme 4* requires the methods from *Themes 1–3*, but with a further step to translate the science to decision-making, and to influence on-the-ground management. From a robust description of population movement, ideally including some analysis of species traits or external factors, it becomes possible to forecast the effects of anthropogenic and environmental changes on future movement corridors or population distributions and to develop conservation and management strategies.

## New ecological insights

Widespread occurrence data and new analytical approaches have allowed scientists to describe large-scale population movement, compare patterns among species or regions, and uncover potentially useful new strategies for conservation in ways that have not always been possible, or may have been very constrained, using only tracking data from focal individuals. Using measures of population-level movement and its correlates, researchers have uncovered broad-scale patterns across species’ annual life cycles, and quantified general relationships between population movement, expansion, or contraction and species traits and environmental associations (Box 3).

**Box 3:** Examples of new ecological insights that have been gained in each thematic area with citations

Ecological insights	
*Research Theme 1—Quantify population-level patterns of movement*	
• Broad scale migration patterns, including looped migration, and directionality [28, 88, 134, 152, 165, 166] • Migration timing, including when animals migrate and how quickly they migrate [47, 92, 105, 155, 167, 168] • Estimates of range expansion or contraction, overall shift in center, area, or edges of range [56, 169]	
*Research Theme 2—Assess how species traits influence population-level movement*	
• Species traits impact range expansion [32] and range shifts during periods of rapid climate change [170] • Species traits (body mass, total migration distance) impact avian migration patterns [28] • Species migratory traits affect sensitivity to migration phenology [171]	
*Research Theme 3—Study how population-level movement correlates with external factors*	
• Distance in range shift relative to temperature change, climatic debt [33], and climate velocity during periods of long-term climate transition [170] • Importance of topography and tailwind for migration [87, 152] • Environmental correlates of migration including atmospheric conditions [172], temperature [62, 154], and ecological productivity [173, 174] • Assess whether species presence or absence across sites is affected by other species presence relative to timing of migration [46]	
*Research Theme 4—Connect movement patterns with conservation or management schema*	
• Association of migratory birds with protected areas and land-cover categories across the annual cycle [175, 176] • Impacts to moving species from projected changes in climate and land use [177] • Impacts to society from range movement or redistribution due to climate change [178] • Potential environmental barriers to migration [153] • Urban effects on occurrence of birds and mammals [179–181] • Conservation planning based on movement and abundance across species’ annual cycles [182]	

## Conclusions and next steps

The rapid growth in occurrence data as well as new computational tools and statistical methods have opened the door to new possibilities for inferring population-level movement across broad spatial scales. While scientists at eBird and those specializing in analysis of WSR data have thus far led the development of datasets and new statistical methods and tools for analyzing movement from occurrence records, many of these approaches could be reasonably adapted to other crowdsourced or occurrence datasets, different taxonomic groups, and other types of research questions addressing population-level movement. Population-level movement studies have previously focused on data from individual studies that represent a relatively small sample of larger populations, but growing networks and increasingly available and interoperable data make larger collaborations and advances possible. Work that integrates different data sources and includes both individual- and population-level movement metrics (e.g., [49, 183–185]) have the potential to create models that share parameters across data, locations, and spatial or temporal resolutions to provide a more comprehensive summary of animal movement. Further, process-based modelling using individual tracking datasets could prove useful for testing hypotheses derived from occurrence data and for developing insights into alternate mechanisms driving observed population-level movement patterns.

As sensor networks for occurrence data mature, current limitations will hopefully be addressed through increased data quantity, improved methods for estimating accuracy and bias, and enhanced metadata standards. Population-level movement studies are “data hungry”—sufficient thresholds of the number of occurrences across space and time are needed to conduct a meaningful analysis—and not all datasets have the appropriate spatiotemporal coverage or volume of records required to make reliable estimates of population movement. Finally, the development of common standards for occurrence datasets could aid in increasing data availability, accessibility, and interoperability, as well as facilitating more robust adjustments for sampling effort and bias. For example, not all crowdsourced data repositories collect the necessary information to account for variation in observer skill or sampling effort, but an endeavor to do so would improve the utility of these data for movement ecology, and other scientific research. In some cases, related parts of sensor networks differ in their data accessibility or the resolution at which key geographic, temporal, or taxonomic variables are recorded, which can make it challenging for researchers to use data across broader spatial scales.

Currently, population movement studies are strongly biased towards birds, followed by other types of aerofauna such as bats and insects, and are geographically biased to North America and Europe. These biases make clear the exciting opportunities for new research in this area. There is high potential for new ecological insights to be gained as data are collected across other parts of the planet and for other taxonomic groups. This review only examined research related to terrestrial animal movement, but many of the same concepts and approaches can be applied to populations of aquatic and marine (e.g., [186, 187]), microbial [29], and plant taxa, which move over generations by natural or human-assisted dispersal (e.g., [188–190]. Using these data and new statistical methods to assess population-level movement can help support current work being carried out with individual tracking to determine how individual movement observations fit within the whole of a population or species’ trajectory and average patterns of movement. In addition, a population-level perspective can help shed new light on large-scale macro-movement patterns and associations with biological and environmental correlates of movement that might not be as obvious when considering the variation between specific individuals that were sampled (Fig. 1). Finally, documenting population movement may help scientists gain a clearer macroecological understanding of species occurrence, range expansion or contraction, migration, and needs for conservation and management in a changing world.

## Data Availability

The datasets used to create figures or examples in the current review are cited within the text and the references and are freely available for download or from the data collector upon reasonable request.
